# Central Dental Association of Northern New Jersey

**Published:** 1897-03

**Authors:** H. S. Sutphen

**Affiliations:** Secretary


### CENTRAL DENTAL ASSOCIATION OF NORTHERN NEW JERSEY.

A regular meeting of the Association was held on Monday evening, October 19, 1896, at the parlors of Mr. Simon Davis, 943 Broad Street, Newark, N. J., the President, Dr. Walter Woolsey, in the chair.

Dr. F. C. Barlow, chairman of the Executive Committee, reported as follows: Your committee announce with pleasure that they have secured for this season the names of more men eminent in our profession than we have been able to procure heretofore, and consequently will have papers of high merit each month. This evening we have the pleasure of introducing to you Dr. Broomell, of Philadelphia, whose lantern slides have been exhibited before many of the colleges, and have elicited encomiums of their merit and accuracy in phases of dental anatomy and histology. The succeeding month we announce as the essayist Dr. Joseph Head, of Philadelphia, who will read a paper on dental ethics. The name of Dr. Head is a sufficient guarantee of the excellent quality of the paper promised. Further announcements will be made later on.

*The President.*—The next thing in order is the paper of the evening. I have the very great pleasure of introducing Dr. I. Norman Broomell, of Philadelphia, who will give us an illustrated lecture on the subjects of dental anatomy, dental pathology, comparative (dental) anatomy, palæontology, and dental histology.

*Dr. Broomell.*—Mr. President and gentlemen, what I have to offer to you to-night will be more in the line of entertainment than of scientific information. The subject will consist of a limited number of slides from each of the following classifications: Dental anatomy and pathology, comparative dental anatomy, palæontology, and dental histology. I might say that a number of these slides I made about two years ago, principally for the purpose of supporting an argument which I had advanced in a paper favoring the half-tone or process work for the illustration of dental subjects in preference to the old but probably more skilful methods of hand engraving. Since that time I have had the satisfaction of seeing a number of the illustrations in what is probably the leading dental journal produced by this method, and it is some satisfaction to me to be informed that my efforts in this direction have had much to do with the change. Until quite recently dental subjects for illustration were not considered to be of such a nature that they could be accurately reproduced by photographic methods.

Some of these slides, as I have said, were made for the purpose of ascertaining what could be done in this direction, while others I have selected from different papers upon the subjects which they respectively represent. I hope the absence of a positive text may not prove a disappointment to you.

[Dr. Broomell then followed with an exhibition of twenty-five slides on anatomy, followed by forty-one on comparative anatomy, also twelve upon histological subjects,—making a total of seventy-eight slides. As these could not be well understood without accompanying illustrations, the following portion from the comparative anatomy of the teeth is given, as representing this interesting collection.—Ed.]

1. Tooth of a mastodon. The elephant is the only living representative of the species known as the mastodon, which lived in a preceding geological epoch. The molar here shown differs from that of an elephant in one or two particulars. The saw-shaped surface does not exist in the elephant's molars, and the lines of enamel are somewhat circular in formation, while in this tooth they are almost parallel. The molars of the elephant are continually in process of destruction and formation, succeeding each other horizontally. They are successively brought forward until each jaw has had on each side six, or twenty-four in all.

2. Palæontology, the science which treats of the evidences of organic life upon the earth during past geological periods. These evidences consist in the remains of plants and animals embedded or otherwise preserved in rocky strata, or upon their surface. The term "fossil," which has been given to these specimens as found, is described as "the remains of an organized body which has been embedded in the earth at an unknown epoch, which has been then preserved, or which has left positive traces of its existence." This excludes, of course, the modern remains of plants or animals which have been buried or lost by landslides, floods, etc., of our times. These evidences may exist in two ways, either the petrified remains themselves, or the imprints, designating the position which they once occupied.

Fragment of upper jaw and teeth from the White River, Dakota. The teeth themselves in this specimen, as in the next few to follow, appear to show no change in structure, while the portion representing the bone has changed into a rock-like mass.

3. Upper first and second true molars from the Niobrara River, Neb.

4. Upper jaw and teeth of extinct mammalia, also found in the Niobrara River, Neb. The buccal aspect of these molars appears to be abraded from the overlapping of the corresponding inferior teeth.

5. Lamna, specimens of the simplest form of shark teeth.

6. Three very good specimens from the White River, Dakota, being of the mastodon family, and depicting a most perfect, petrified mass, even to the striated condition peculiar to rock-formation.

7. A tooth from the extensive marine family, the shark, coextensive with the rays and skates. In the earlier geological times the shark attained tremendous proportions, frequently being from thirty to forty feet in length. The tooth specimen here shown represents one of the large variety, but of the simple form, the inner cutting edge being only slightly serrated, while the outer edge is a knife-cutting one. In some of the tertiary-formations at Malta, teeth of this class have been found measuring seven inches in length and four and one-half inches in breadth at the base.

8. Another variety of shark tooth, and a typical tooth of a large proportion of the shark family, conical in shape, with sharp and deeply-serrated edges, and a separate cusp upon either side at the gum margin. These teeth are placed in the jaws in several rows, and the anterior ones have erectile power. Some genera have the peculiarities of a mesial tooth, and when this centre tooth does not exist there is left an unoccupied space of about the size of the adjoining teeth. The deeply concave surface rests upon the jaw, while the erectile muscles are attached upon either side.

9. Teeth of extinct miocene mammalia, found in the marl banks of Cumberland County, N. J. The formation of the enamel of these teeth is peculiar, being one mass of sharply-defined, conical-shaped cusps, not shown very distinctly, however, in the picture.

10. Dental pavement. The petrified remains of the pavement-like teeth of one of the extinct shark family. Fishes provided with teeth of this form use them by a rubbing or triturating motion.

11. Here is shown another set of teeth of the same order, belonging to the ray family, each being provided with two sets, and when actively engaged in performing their duty they rotate or rock back and forth upon each other, crushing the sharp, penetrating bones of their prey, without fear of injury to themselves.

12. Two more specimens, showing not only a peculiar formation, but a neat and almost beautiful arrangement of the pavement-like dentures, belonging to the fossil fish of the skate family. At one point you may see one of these teeth which has been removed from its shallow socket, while in the other specimen may be seen the very beautiful arrangement of the enamel.

13. Another specimen of the pavement-like type, the teeth occupying the whole surface of the maxillary bone.

14. Crustacean, Phillippi, Japan. Port Jackson shark.

15. Elephant tusks. The tusks of the elephant are nothing less than monstrous incisor teeth. The temporary tusks make their appearance about the sixth month, and are not more than two inches in length and one-third of an inch in diameter. These are shed about the second year, and the permanent tusks begin to grow, continuing to do so from their base during the life of the animal.

On motion of Dr. Meeker a vote of thanks was extended to Dr. Broomell for his excellent entertainment.

Adjourned.

H. S. SUTPHEN, D.D.S.,
*Secretary.*

